# Missed opportunities for risk reduction: type 1 diabetes management in older adults in DPV and T1DX-QI registries

**DOI:** 10.1210/jendso/bvag007

**Published:** 2026-01-19

**Authors:** Kathryn L Fantasia, Stefanie Lanzinger, Saketh Rompicherla, Jennifer J Grammes, Grenye O’Malley, Julia K Mader, Lauren Golden, Florian Kopp, David M Maahs, Peter M Jehle, Osagie Ebekozien, Reinhard W Holl

**Affiliations:** Department of Medicine, Section of Endocrinology, Diabetes, Nutrition and Weight Management, Boston University Chobanian and Avedisian School of Medicine, Boston, MA 02118, USA; Department of Medicine, Evans Center for Implementation and Improvement Sciences, Boston University Chobanian and Avedisian School of Medicine, Boston, MA 02118, USA; CAQM, University of Ulm, Ulm 89081, Germany; German Center for Diabetes Research (DZD), Munich-Neuherberg 85764, Germany; T1D Exchange, Boston, MA 02110, USA; Health Psychology, Johannes Gutenberg University, Mainz 55099, Germany; Department of Medicine, Division of Endocrinology, Icahn School of Medicine at Mount Sinai, New York, NY 10029, USA; Department of Internal Medicine, Division of Endocrinology and Diabetology, Medical University of Graz, Graz 8036, Austria; Center for Diabetes and Metabolic Health, New York University Langone Health, New York, NY 10017, USA; Diabetes Center, University of Augsburg, Augsburg 86159, Germany; Department of Pediatrics, Division of Endocrinology, Stanford University, Stanford, CA 94304, USA; Department of Internal Medicine, Martin-Luther-University, Halle-Wittenberg, Lutherstadt Wittenberg 06886, Germany; T1D Exchange, Boston, MA 02110, USA; CAQM, University of Ulm, Ulm 89081, Germany; German Center for Diabetes Research (DZD), Munich-Neuherberg 85764, Germany

**Keywords:** cardiovascular disease, cross-sectional study, diabetes technology, geriatrics, type 1 diabetes mellitus

## Abstract

**Aims:**

To examine prescription of guideline-recommended therapies and achievement of treatment targets across the span of older adulthood in type 1 diabetes (T1D) in the United States and Germany/Austria.

**Materials and Methods:**

Cross-sectional data of adults aged ≥60 years with T1D for ≥1 year seen in 2022 in the T1D Exchange Quality Improvement Collaborative (T1DX-QI) and the Diabetes Prospective Follow-up (DPV) registry. Descriptive statistics and within-registry comparisons across age groups using analysis of variance and chi-squared tests were used to analyze the data.

**Results:**

Thirty-six hundred adults aged ≥60 years, median age 67.5 [interquartile range (IQR) 63.4, 72.8] in T1DX-QI (n = 1549) and 68.9 (IQR 63.6, 75.7) in DPV (n = 2051) were included. The prevalence of atherosclerotic cardiovascular disease (ASCVD) (34.6% vs 16.8%) and chronic kidney disease (28.5% vs 11.8%) was higher in the DPV than the T1DX-QI. Lipid-lowering therapy for secondary prevention (52.9% vs 38%) and angiotensin-converting enzyme inhibitor/angiotensin receptor blocker use (55.3% vs 44.8%) were higher in the DPV. Continuous glucose monitoring use was similar (50.3% vs 47.9%), insulin pump use was >2 × higher (40.7% vs 17%), and automated insulin delivery use was >3 × higher (20.4% vs 6.4%) in the T1DX-QI as compared to the DPV.

**Conclusion:**

Despite a high prevalence of ASCVD and risks of hypoglycemia, guideline-recommended treatments including lipid-lowering therapy for secondary prevention and diabetes technologies were used in approximately half or fewer of older adults with T1D. Additional attention to prescribing and practices to support clinicians and older adults in the use of diabetes technologies is urgently needed.

The prevalence of type 1 diabetes mellitus (T1D) in adults 60 years of age and older is increasing due to both an increase in incident cases and improved life expectancy [1-3]. By 2040, the population with T1D is expected to approximately double among those 70 and older in high-income countries as compared to 2021 prevalence estimates [3]. However, despite recent advances in diabetes management, there remain significantly higher mortality rates and a greater than 10-year gap in life expectancy for people with T1D as compared to those without diabetes [3, 4].

Cardiovascular and renal disease account for the majority of excess mortality in T1D and increase in prevalence with age [5, 6]. Older adults with T1D face additional health risks associated with a higher burden of hypoglycemia and its sequelae, including increased risks of falls and fractures, worse cognition, dementia, and cardiovascular events [7-11].

Multiple studies have demonstrated the impact of glycemia, treatment of hypercholesterolemia, and blood pressure management on cardiovascular and renal outcomes for those living with diabetes [12]. Though older adults were often excluded from initial pivotal device trials, subsequent studies have demonstrated benefits of reduced hemoglobin A1c (HbA1c) and less hypoglycemia for older adults using advanced diabetes technologies, including continuous glucose monitoring (CGM) [13], insulin pumps [14], and automated insulin delivery (AID) systems [15].

Despite the proven benefits of treatments for the prevention of cardiovascular events and renal disease progression, varying rates of management of cardiovascular risk factors in T1D are seen. Benchmarking international practice patterns across established T1D registries provides an opportunity to identify practice gaps for future intervention and assess their impact. Prior studies from the T1D Exchange (T1DX) and the German/Austrian Diabetes Patient Follow-up registry (DPV) have demonstrated low to moderate rates of prescription of cardiorenal risk-reducing therapies across the lifespan [16, 17]. Though rates of prescriptions for advanced diabetes technologies have increased as evidence for glycemic and quality of life benefits has grown, updated rates of use among older adults are unclear. Further, studies on care delivery and outcomes amongst older adults with T1D are limited and often do not differentiate data for those beyond 60 years of age.

In the setting of a growing population of older adults with T1D, and in light of evidence of undertreatment of cardiovascular disease in older adults [18] and higher rates of undertreatment with increasing age [19], this study aimed to (1) describe and compare treatments and complications in older adults from the US T1DX-QI and the European DPV registry and (2) examine receipt of guideline-recommended care including diabetes devices and cardiorenal risk reducing therapies in relation to the achievement of treatment targets across the span of older adulthood.

## Methods

This cross-sectional study examined data from the T1DX-QI and DPV registry.

### Data sources

Established in 2016 by the T1D Exchange to improve care delivery for people with T1D, the T1DX-QI clinic network is a learning collaborative of 21 adult endocrinology clinics across the United States who care for >100 000 people with T1D. The T1DX-QI partners with participating centers to collect standardized data on diabetes care and outcomes for benchmarking and centralized analysis [20]. All participating centers comply with their local institutional review board regulations for the use of protected health information. The DPV registry was established in 1995 at the University of Ulm, Germany. Inclusive of 485 specialized diabetes care centers in 2022, diabetes departments in university hospitals, community-based general hospitals, and rehabilitation units across Germany, Austria, Switzerland, and Luxembourg, the DPV collects anonymized data for central analyses and benchmarking (https://buster.zibmt.uni-ulm.de/projekte/DPV/ for more information). The DPV initiative has been approved by the ethics committee at the University of Ulm (314/21) and by the local review boards at each participating site. In contrast to prior studies where comparison between DPV and the T1DX registry was possible due to a data use agreement, sharing of the T1DX-QI data is not currently permitted.

### Study population

Adults in the T1DX-QI and DPV registry ≥60 years of age with T1D duration ≥1 year who had data collected during calendar year 2022 were included in this study. Adults age ≥60 years and older were included, consistent with the United Nations definition of an older adult [21].

### Variables

Demographic data, HbA1c, body mass index, systolic and diastolic blood pressure, lipid and creatinine values, micro and macrovascular complications (retinopathy, neuropathy, albuminuria status, chronic kidney disease (CKD), atherosclerotic cardiovascular disease (ASCVD), prescription data (insulin regimen, lipid-lowering therapy, antihypertensive use), device use (CGM, insulin pump, AID), and tobacco use are collected from medical records. Race and ethnicity are not collected in the DPV registry and are therefore not included in this analysis.

Hypertension was defined as blood pressure ≥140/90 mmHg or treatment with antihypertensive medication. Dyslipidemia was defined as low-density lipoprotein (LDL) >130 mg/dL or prescription for lipid-lowering therapy. Participants were identified as having overweight or obesity if their body mass index was ≥25 or ≥30 kg/m^2^, respectively. Estimated glomerular filtration rate (eGFR) was calculated using the chronic kidney disease epidemiology collaboration 2021 race-free equation for adults [22]. CKD was defined as eGFR ≥60 mL/min/1.73m^2^ with microalbuminuria (urine albumin 30-300 mg/g creatine) or eGFR <60 mL/min/1.73 m^2^, and end-stage renal disease was defined as eGFR <15 mL/min/1.73 m^2^, history of kidney transplantation, or dialysis. ASCVD was defined as having any of the following based on International Classification of Diseases, Tenth Revision (ICD-10) codes: myocardial infarction, unstable angina, stable angina, history of coronary revascularization, stroke, transient ischemic attack, or peripheral arterial disease. Retinopathy and neuropathy were defined as an exam demonstrating clinical findings and/or ICD-10 code. A history of retinopathy or neuropathy was included to account for missing data related to eye exams every other year. Diabetic ketoacidosis (DKA) was defined as hospital admission for DKA and severe hypoglycemia (SH) as blood sugar <54 mg/dL or low blood sugar requiring assistance. Both were identified from medical records and are reported as rate per 100 person-years. T1DX-QI- and DPV-specific definitions are in Table S1 [23]. Lipid-lowering therapy included statins, ezetimibe, fibrates, bempedoic acid, and proprotein convertase subtilisin/kexin type 9 inhibitors; antihypertensive medications included angiotensin-converting enzyme inhibitors (ACE-i), angiotensin receptor blockers (ARBs), aldosterone receptor antagonists, α blockers, α central agonists, β blockers, calcium channel blockers, diuretics, renin inhibitors, and vasodilators. See Tables S1 to S3 [23] for measure definitions, medication lists, and ICD-10 codes for diabetes-related complications (Open Science Framework https://doi.org/10.17605/OSF.IO/VNZHG) [23].

Treatment goals and targets for blood pressure as well as recommendations for the use of cardiorenal risk reducing medications (statins, ACE-i, or ARBs) were aligned with the American Diabetes Association 2022 Standards of Care [24, 25], the European Association for the Study of Diabetes, and American Diabetes Association 2021 consensus report [26] as these were the most recent clinical practice guidelines relevant to the care of adults with T1D seen in the United States and Europe at the time. Blood pressure <140/90 mmHg and LDL <70 mg/dL for individuals with ASCVD were identified as meeting target goals for treatment, in alignment with most clinical practice guidelines available in 2022. Additional analyses were performed for blood pressure <130/80 mmHg in alignment with the 2017 American College of Cardiology/American Heart Association guidelines [16].

### Statistical analysis

Demographic and clinical characteristics were calculated using descriptive statistics and are presented as median and interquartile range for continuous data or percentages for categorical variables. Age was categorized into 3 groups: 60 to <70 years, 70 to <80 years, and ≥80 years. Within the T1DX-QI and DPV registry, comparisons across age groups were conducted using ANOVA and chi-squared tests. Rates of ACE-i/ARB prescription were analyzed according to the presence of hypertension and proteinuria. Lipid-lowering therapy prescription was analyzed among those with or without ASCVD. No statistical comparison tests were performed between T1DX-QI and DPV as all analyses were conducted for each registry separately as per the registries' data-sharing and privacy guidelines. All tests were 2-sided (α=.05). Analyses were performed in SAS statistical software version 9.4 TS1M8 (SAS Institute, Cary, NC, USA) and R software, version 4.3.2 (R Foundation for Statistical Computing). Results are reported in accordance with the STROBE reporting guideline [27].

## Results

### Description of populations

Due to data-sharing restrictions, no statistical comparison tests were performed between the 2 registries. The final study population included a total of 3600 adults aged 60 and older: 1549 individuals from T1DX-QI and 2051 from DPV. Demographic data and clinical characteristics by registry are shown in Table 1. The median age was 67.5 years [interquartile range (IQR) 63.4, 72.8] in T1DX-QI and 68.9 years (IQR 63.6, 75.7) in the DPV. Overall, the T1DX-QI population had a lower median HbA1c (7.1%, 54.4 mmol/mol vs 7.4%, 57.9 mmol/mol) than the DPV population. There were higher rates of obesity (28.8% vs 20.8%), similar median blood pressure, and lower median LDL cholesterol (76.5 mg/dL vs 96 mg/dL) in the T1DX-QI than in the DPV registry. Rates of proteinuria were more than twice as high (58.3% vs 27.7%), and rates of retinopathy (19.9% vs 32.4%) and neuropathy (29.7% vs 68.3%) were lower in the T1DX-QI than in the DPV registry.

**Table 1:** Participant characteristics

	% missing data		T1DX-QI n = 1549	DPV n = 2051
	TIDX-QI	DPV		
Sex, female	0	0	797 (51.5)	978 (47.7)
Age, years (median, IQR)	0	0	67.6 (63.5, 72.9)	68.9 (63.6, 75.7)
HbA1c (median, IQR)	16.1	3.9		
%			7.1 (6.5, 7.9)	7.4 (6.8, 8.2)
mmol/mol			54.4 (47.5, 62.8)	57.9 (50.6, 66.1)
BMI kg/m^2^ (median, IQR)	14	5	26.8 (23.7, 30.8)	25.9 (23.2, 29.3)
Obesity (BMI ≥ 30)	14	5	383 (28.8)	405 (20.8)
Underweight (BMI <18.5)	14	5	28 (2.1)	49 (2.5)
LDL cholesterol mg/dL (median, IQR)	86.4	31.3	76.5 (60, 93)	96 (73, 122)
BP mmHg (median, IQR)	16.2	5.2		
Systolic			132 (122, 146)	135 (125, 147)
Diastolic			74 (68, 79)	78 (70, 81)
Albuminuria	88.8	49.9		
Urine albumin 30-300 mg/g Cr			80 (46.2)	229 (22.3)
Urine albumin >300 mg/g Cr			21 (12.1)	55 (5.4)
History of retinopathy	0	54.4	308 (19.9)	303 (32.4)
History of neuropathy	0	0	460 (29.7)	1401 (68.3)
ASCVD	0	0	260 (16.8)	710 (34.6)
Hyperlipidemia	0	22.5	913 (58.9)	1085 (68.3)
Tobacco use (current)	65.1	38.7	40 (7.4)	194 (15.4)
Hypertension				
BP ≥140/90 mmHg	0	4	952 (61.5)	1368 (69.5)
BP ≥130/80 mmHg	0	4	1072 (69.2)	1596 (81.1)
Chronic kidney disease	0	0	183 (11.8)	640 (31.2)
DKA (rate per 100 person-years)			2.2	4.56
Severe hypoglycemia (rate per 100 person years)			2.7	12.14
Lipid-lowering therapy	0	0	722 (46.6)	923 (45)
ACE-i/ARB	0	0	714 (46.1)	1134 (55.3)
CGM use	0	0	889 (50.3)	954 (46.5)
Insulin pump use	0	0	537 (40.7)	365 (17.8)
AID use	47.8	0	165 (20.4)	125 (6.1)

Data presented as n (%) unless otherwise indicated.

Abbreviations: ACE-i, angiotensin-converting enzyme inhibitors; AID, automated insulin delivery; ARB, angiotensin receptor blockers; ASCVD, atherosclerotic cardiovascular disease; BMI, body mass index; BP, blood pressure; CGM, continuous glucose monitoring; DKA, diabetic ketoacidosis; DPV, Diabetes Prospective Follow-up; HbA1c, hemoglobin A1c; IQR, interquartile range; LDL, low-density lipoprotein; T1DX-QI, T1D Exchange Quality Improvement Collaborative.

**Figure 1:**
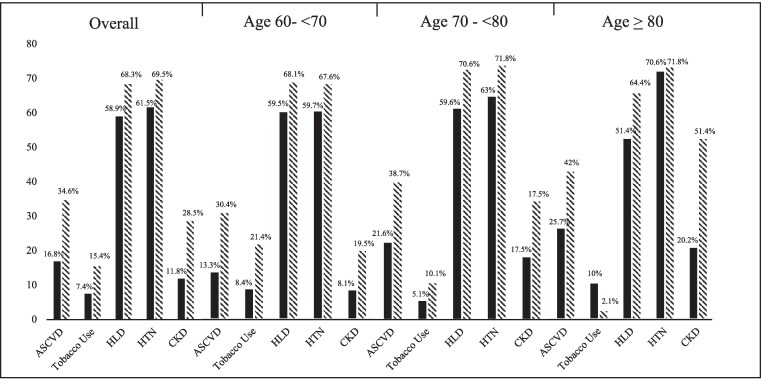
Prevalence of established cardiovascular disease and cardiovascular risk factors stratified by age. Percentage with ASCVD, current tobacco use, HLD, HTN, and CKD in each registry, stratified by age. Tobacco use data available for 542 participants in the T1DX-QI. Solid black bars represent T1DX-QI and striped bars represent DPV. Abbreviations: ASCVD, atherosclerotic cardiovascular disease; CKD, chronic kidney disease; DPV, Diabetes Prospective Follow-up; HLD, hyperlipidemia; HTN, hypertension; T1DX-QI, T1D Exchange Quality Improvement Collaborative.

The prevalence of established cardiovascular disease and cardiovascular risk factors by registry overall and stratified by age group is shown in Fig. 1. Rates of established ASCVD were higher overall (30.4% vs 16.8%) and across age groups in the DPV than the T1DX-QI. Across both the T1DX-QI and DPV, rates of ASCVD and CKD were significantly higher with increasing age (*P* < .001) and were higher in the DPV than in the T1DX-QI. Rates of hyperlipidemia and hypertension were prevalent in over half of participants across registries and were not statistically significantly different across age groups. Rates of current tobacco use were twice as high in the DPV registry overall as compared to the T1DX-QI (15.4% v 7.4%), and while rates of tobacco use decreased in increasing age groups in the DPV registry (21.4% vs 2.1%; *P* < .001), they were highest in those ≥80 years in the T1DX QI (10%) and lowest in this age group in the DPV (2.1%).

### Receipt of guideline-recommended therapies and achievement of treatment targets

The proportion of participants receiving guideline-recommended cardiovascular and renal risk-reducing therapies and achieving treatment targets overall and stratified by age group is shown in Table 2. Among individuals without established ASCVD, lipid-lowering prescription for primary prevention was 47.9% in the T1DX-QI and 40.8% in the DPV. In the T1DX-QI, the rate of prescription for lipid-lowering therapy for primary prevention was 50.4%, 47.2%, and 43.8% for ages 60 to <70, 70 to <80, and ≥80, respectively. In the DPV, the rate of prescription for lipid-lowering therapy for primary prevention was 41.5%, 43.1%, and 33% for ages 60 to -<70, 70 to <80, and ≥80, respectively. However, these differences across age groups were not statistically significant. For individuals with established ASCVD, lipid-lowering therapy prescription for secondary prevention was lower than that for primary prevention in the T1DX-QI (35.4% vs 47.9%, respectively) and higher in the DPV cohort (52.9% vs 40.8%, respectively). Among those with established ASCVD, the proportion achieving LDL treatment targets was low: only approximately one-third (36.5%) in the T1DX-QI and just over one-quarter (27.8%) in the DPV met guideline-recommended LDL treatment targets of <70 mg/dL. Greater proportions of those with ASCVD had LDL <100 mg/dL in both registries. Within both cohorts, among those with ASCVD, those aged 70 to <80 years had the highest rates of meeting LDL treatment goals; this difference was statistically significant in the DPV registry (*P* = .003).

**Table 2:** Proportion receiving guideline-recommended care and achieving treatment targets

	T1DX-QI					DPV				
	Overall (n = 1549)	Age 60-<70 (n = 967)	Age 70-<80 (n = 473)	Age ≥80 (n = 109)	*P* value	Overall (n = 2051)	Age 60-<70 (n = 1138)	Age 70-<80 (n = 594)	Age ≥ 80 (n = 319)	*P*-value
Lipid-lowering therapy for primary prevention*^a^*	630/1314 (47.9)	420/844 (50.4)	175/389 (47.2)	35/81 (43.8)	.4	547/1341 (40.8)	329/792 (41.5)	157/364 (43.1)	61/185 (33)	.12
Lipid-lowering therapy for secondary prevention*^b^*	92/260 (35.4)	49/129 (38)	33/102 (33)	10/29 (34.5)	.6	375/709 (52.9)	173/345 (50.1)	133/230 (57.8)	69/134 (51.5)	.46
ACE-i/ARB*^c^*	714/1549, (46.1)	433/967 (44.8)	219/473 (46.3)	62/109 (56.9)	.06	1134/2050 (55.3)	601/1137 (52.9)	337/594 (56.7)	196/319 (61.4)	.005
LDL										
<70 mg/dL*^d^*	77/211 (36.5)	52/147 (35.4)	21/50 (42)	4/14 (28.6)	.6	142/510 (27.8)	62/245 (25.3)	53/169 (31.4)	27/96 (28.1)	.003
<100 mg/dL	168/211 (79.6)	118/147 (80.3)	39/50 (78)	11/14 (78.6)	.9	304/510 (59.6)	141/245 (57.6)	104/169 (61.5)	59/96 (62.5)	.002
BP <140/90mmHg	852/1298 (65.6)	546/810 (67.4)	253/399 (63.4)	53/89 (59.7)	.2	668/1110 (60.2)	375/589 (63.7)	175/328 (53.4)	118/193 (61.1)	.2
BP <130/80mmHg	388/1298 (29.9)	239/810 (29.5)	122/399 (30.6)	27/89 (30.3)	.6	392/1110 (35.3)	214/589 (36.3)	103/328 (31.4)	75/193 (38.9)	.3

Data are presented as numerator/denominator and percent.

Abbreviations: ACE-i, angiotensin-converting enzyme inhibitors; ARB, angiotensin receptor blockers; BP, blood pressure; DPV, Diabetes Prospective Follow-up; LDL, low-density lipoprotein; T1DX-QI, T1D Exchange Quality Improvement Collaborative.

^*a*^Primary prevention indicates individuals without established atherosclerotic cardiovascular disease.

^*b*^Secondary prevention indicates individuals with established atherosclerotic cardiovascular disease.

^*c*^Among those with hypertension and proteinuria.

^*d*^Among those with atherosclerotic cardiovascular disease.

ACE-i or ARB use among those with hypertension and proteinuria was higher in the DPV than in the T1DX-QI (55.3% vs 46.1%) with higher rates of use with increasing age groups and the highest rates of use among those ≥80 years of age. This difference was statistically significant in the DPV registry (*P* = .005). For those with hypertension, approximately two-thirds of those in T1DX-QI (65.6%) and in the DPV (60.2%) met recommended blood pressure targets of systolic blood pressure <140 mmHg and diastolic blood pressure <90 mmHg. In both cohorts, the proportions meeting blood pressure targets were lower among those ≥70 than those <70 years, despite higher rates of ACE-i/ARB use in increasing age groups.

### Device use and glycemia

**Figure 2:**
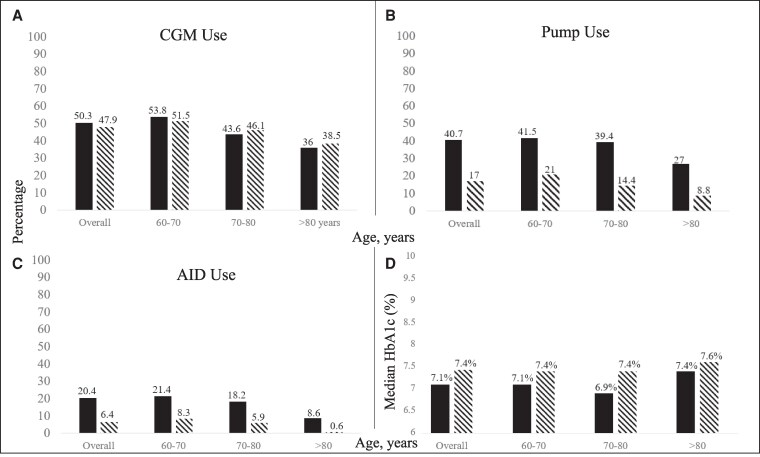
Device use and glycemia across registries. (A) Percentage using CGM in each registry, stratified by age group. (B) Percentage using pump in each registry, stratified by age group. (C) Percentage using AID in each registry, stratified by age group. (D) Median HbA1c in each registry, stratified by age group. Solid black bars represent T1DX-QI and striped bars represent DPV. Abbreviations: AID, automated insulin delivery; CGM, continuous glucose monitoring; DPV, Diabetes Prospective Follow-up; HbA1c, hemoglobin A1c; T1DX-QI, T1D Exchange Quality Improvement Collaborative.

Overall, CGM use was similar, insulin pump use was approximately twice as high, and AID use was approximately 3 times higher in the T1DX-QI as compared to the DPV. Device use was lower with increasing age across both registries, with the lowest rates of device use seen in adults >80 years of age (all *P* < .001 in DPV) (Fig. 2). The median HbA1c was lower in the T1DX-QI than the DPV registry overall and across age groups (Fig. 1). Rates of DKA were lower in the T1DX-QI as compared to the DPV registry (2.2 vs 4.56 events per 100 person-years, respectively). Rates of SH were also lower in the T1DX-QI as compared to the DPV (2.7 vs 12.14 events per 100 person-years, respectively).

## Discussion

In this transatlantic comparison of the care of older adults with T1D, we identified that within a population at high risk for diabetes-related morbidity and mortality, prescription of guideline-recommended risk-reducing medications and devices were seen in approximately half or fewer of patients, and achievement of treatment goals for hypertension and hyperlipidemia were seen in two-thirds or fewer. Notably, lipid-lowering therapy for secondary prevention was observed in only 53% of the DPV cohort and 35% of the T1DX-QI cohort, and one-third or fewer of these patients achieved guideline-recommended LDL targets. Further, therapies that may mitigate hypoglycemia, including CGM and AID, were underused. Only half of the participants overall were prescribed CGM with the lowest rates in those 80 years of age and older (36% T1DX-QI and 28.5% DPV). Rates of AID use were much lower, with 20% overall in the T1DX-QI and 6% in the DPV. Like with CGM, the lowest rates of use were seen among those 80 years and older (<10% in T1DX-QI and <1% in DPV).

Multiple professional society guidelines in both the United States [24, 25, 28] and Europe [26, 29] recommend use of therapies with proven cardiorenal risk reduction benefits, including ACE-i/ARB and lipid-lowering agents for adults with diabetes. Though fewer than 25% of people in the United States with diabetes meet all treatment goals for glycemia, blood pressure, and cholesterol [30], prior studies of treatment of cardiovascular risk factors in T1D demonstrated that among older adults seen between 2016 and 2018, 50% or more met treatment goals for blood pressure and lipids [16] and 40% to 60% of older adults in the DPV and T1DX registry seen in 2011 and 2012 were on statin therapy [17]. In contrast, in our study, we demonstrated that only 35% to 53% of adults 60 years and older were on guideline-recommended lipid-lowering therapy. Notably, rates of lipid-lowering therapy prescription for primary prevention were higher than for secondary prevention in the T1DX-QI. Rates of lipid-lowering therapy prescription for secondary prevention were higher than for primary prevention in the DPV, following an expected pattern.

The differences in the prescribing pattern observed for lipid-lowering therapy, including lower rates of lipid-lowering therapy prescription in 2022 cohorts as compared to earlier studies, may be multifactorial. First, the US-based T1DX-QI cohort examined in this study includes patients seen in T1DX-QI participating centers and therefore reflects a broader cohort of patients than those seen in prior studies evaluating the T1DX registry. Next, while guidelines recommend lipid-lowering therapy for adults aged ≤75, age-specific guidelines and benefits of lipid-lowering therapies for primary prevention in adults 75 or older are unclear [31]. Finally, though we are unable to directly assess individual-level longitudinal changes in prescriptions, increased emphasis on deprescribing (ie, the discontinuation of potentially inappropriate medications with the goal of managing polypharmacy), including as part of the “4Ms” framework for care of older adults developed in 2017 [32], may contribute to lower rates of lipid-lowering therapy as compared to prior studies. However, among those with a history of ASCVD, there has been longstanding, strong evidence for the benefit of use of lipid-lowering therapy in older adults [33], and statin therapy for primary prevention for adults older than 75 years is recommend by US professional organizations including the National Lipid Association and the American Geriatrics Association, with recommendations for deprescription in select patients with life-limiting diseases [34]. Despite this, only 35% in the T1DX-QI and 53% in the DPV were prescribed lipid-lowering therapy and only 36.5% in the T1DX-QI and 27.8% in the DPV met the LDL target <70 mg/dL [28]. Our finding of low rates of lipid-lowering therapy for secondary ASCVD prevention in older adults is consistent with prior literature demonstrating decreasing likelihood of statin prescription with increasing age despite high baseline risk [33]. These lower-than-recommended rates of prescription of cardiorenal risk-reducing therapies in older adults with T1D may reflect trends in deprescribing and/or beliefs about benefits of such therapies in an older population who may experience multimorbidity and polypharmacy. Variation in prescribing of lipid-lowering therapy and ACE-i/ARB between the US- and European-based cohorts may reflect regional differences in clinician beliefs about and patient preferences related to deprescribing [35]. While prescription of these therapies should be consistent with preferences and comorbidities in older adults, given the strong evidence for benefit of lipid-lowering therapy for secondary prevention in particular, this may not reflect appropriate deprescribing as those with life-limiting diseases are unlikely to constitute most older adults with T1D in the T1DX-QI and DPV cohorts. Further, deprescribing lipid-lowering therapy has not been studied in older adults with T1D, a population with high cardiovascular disease risk and burden. Given this, increased attention to prescribing cardiovascular risk reducing therapies in older adults is warranted.

Despite demonstrated benefits of CGM in older adults, including reduced hypoglycemia and improved quality of life [36], use was seen in half or less of those in this study, and rates were relatively similar across registries. As compared to prior studies reporting on device use in older adults from the T1DX and DPV from 2011 to 2012 [17] and 2016 to 2018 [16], as well as DPV specific data from 2008 to 2018 [37] and 2019 to 2021 [38], rates of CGM and insulin pump/AID use were higher in this study. This study demonstrates that compared to previously published data from 2016 to 2018 [16], rates of CGM use among adults ≥65 years of age have increased nearly 5-fold in the DPV (47.9% vs 9%) and have doubled in the T1DX-QI (50.3% vs 26%), whereas rates of insulin pump use overall (inclusive of AID use) have remained relatively stable [16]. Despite this substantial increase in CGM use in older adults, rates of device use in older adults remain lower than in pediatric populations, including children <6 years of age [39]. Prior data from the T1DX registry have demonstrated that the largest increases in rates of diabetes technology use have been in children, with a 10-fold increase in CGM use among those under 6 years of age between 2010 and 2018 [40]. In contrast to our data on older adults, CGM use in patients <25 years of age doubled from 2017 to 2020 to 49% in the T1DX-QI and 76% in the DPV [41].

To our knowledge, this is the first study to report on AID use across the span of older adulthood. Though rates of AID use were higher in the T1DX-QI than in the DPV, lower rates of device use with increasing age were seen across both registries. The lowest rates of AID use were seen in adults ≥80 years. Despite higher rates of insulin pump and AID use in the T1DX-QI, rates of DKA were lower than in the DPV. Reasons for the higher rate of AID use in the T1DX-QI as compared to the DPV may include that the T1DX-QI only collects data from endocrine specialty centers whereas the DPV registry includes data from all healthcare sites. While a discussion of health policy and insurance is beyond the scope of this paper, coverage for devices is available through Medicare for older adults ≥65 years with T1D in the United States and is a covered benefit in Germany, Austria, and Luxembourg and therefore does not explain the lower rates of use. The trend of decreasing rates of device use in increasing age groups may reflect perceptions of increased burden from device use. Prior studies have demonstrated that perceived burden of CGM is highest in those over the age of 50 [42]. However, recent studies of the experience of older adults using AID indicate that AID is the preferred insulin delivery method [43]. Additionally, decreased rates of use may reflect beliefs about the ability of older adults as compared to younger adults to navigate the use of AID systems [44] and/or beliefs about potential benefits of AID in this population despite evidence from both randomized-controlled trials and real-world evidence that AID systems improve glycemia and reduce hypoglycemia in older adults [15, 45]. While ascertainment bias may contribute to the lower rates of SH seen in the T1DX-QI, it is also possible that the higher rates of diabetes technology use contribute to the lower rates of SH seen in the T1DX-QI and suggest that, among older adults, a greater proportion of the population would benefit from use. Considering the high burden and risks of hypoglycemia in older adults, additional studies to understand determinants of device use for older adults, including clinician perspectives on use of AID in older adults, and strategies to increase use among those who are interested in their use, are required.

Reasons for lower rates of risk-reducing medications, specifically lipid-lowering therapy in those with ASCVD and ACE-i/ARB use despite high rates of microalbuminuria, and higher rates of device prescribing in the T1DX-QI as compared to the DPV are unclear. However, this pattern may reflect recent emphasis on increasing device use within the T1DX-QI, perhaps at the expense of continued focus on other aspects of guideline-recommended interventions. Taken together, though rates of device use in older adults have increased from prior studies, these findings raise concern for ageism (discrimination against an individual on the basis of age) as a factor contributing to underprescribing in T1D. Ageism is pervasive and associated with worse health outcomes [46]. Specifically, the lack of appropriate use of lipid-lowering therapy in a large proportion of older adults with ASCVD and both the overall lower rates of device use and decreasing rates of use with increasing age in this population as compared to overall rates of device use in T1D [47] is concerning. Older age has been demonstrated to impact diagnostic evaluations and prescribing practices and is associated with underprescription of beneficial therapies [19].

The strengths of this study include the evaluation of a large cohort of older adults with T1D and examination of care across the spectrum of age for adults 60 years and older, an understudied and rapidly growing population. However, given the observational, cross-sectional nature of the study, data should be interpreted with caution. As data are collected from the electronic medical record, missing data for certain variables (more prevalent in the T1DX-QI) likely contribute to discrepant findings related to proteinuria and CKD and represent a significant limitation; as such, data with high rates of missingness (including lipids, albuminuria, and retinopathy) should be interpreted with caution. Additionally, ascertainment bias and underreporting of complications and comorbidities may be possible. In particular, SH may be underreported. Diabetes duration data is not included due to constraints in obtaining this data from real-world electronic medical record data. Further, as data on cognitive decline and frailty were not available, the impact of these factors on device prescribing could not be assessed, nor could the impact of socioeconomic status or race/ethnicity be evaluated as these data are not collected by the T1DX-QI and DPV registry, respectively. While the T1DX-QI has established benchmarks for T1D management and outcomes in the United States, the T1DX-QI only includes diabetes specialty centers and is not population based. In a recent analysis of Medicare claims, only one-third of older adults with T1D in the United States received office-based endocrinology specialist care [48]. Given that rates of device use are higher among those seen in endocrine specialty centers as compared to primary care [49], it is likely that these data are overestimates of the general population of older adults with T1D.

As the population of older adults with T1D increases, additional research to identify contributors to underprescribing of beneficial therapies and to determine best practices for supporting older adults and their caregivers with use of diabetes technology is necessary. Increased attention to prescribing practices for older adults is warranted to ensure that those at high risk for cardiovascular events and hypoglycemia and its sequelae receive evidence-based treatment.

## Data Availability

Restrictions apply to the availability of some or all data generated or analyzed during this study to preserve patient confidentiality or because they were used under license. The corresponding author will on request detail the restrictions and any conditions under which access to some data may be provided.

## Abbreviations

ACE-i: angiotensin-converting enzyme inhibitors
AID: automated insulin delivery
ARB: angiotensin receptor blocker
ASCVD: atherosclerotic cardiovascular disease
CGM: continuous glucose monitoring
CKD: chronic kidney disease
DKA: diabetic ketoacidosis
eGFR: estimated glomerular filtration rate
ICD-10: International Classification of Diseases, Tenth Revision
LDL: low-density lipoprotein
SH: severe hypoglycemia
T1D: type 1 diabetes
